# Machine Learning-Mediated Development and Optimization of Disinfection Protocol and Scarification Method for Improved In Vitro Germination of Cannabis Seeds

**DOI:** 10.3390/plants10112397

**Published:** 2021-11-06

**Authors:** Marco Pepe, Mohsen Hesami, Andrew Maxwell Phineas Jones

**Affiliations:** Department of Plant Agriculture, Gosling Research Institute for Plant Preservation, University of Guelph, Guelph, ON N1G 2W1, Canada; pepem@uoguelph.ca (M.P.); mhesami@uoguelph.ca (M.H.)

**Keywords:** hydrogen peroxide, sodium hypochlorite, generalized regression neural network, genetic algorithm, scarification, seed dormancy, plant tissue culture

## Abstract

In vitro seed germination is a useful tool for developing a variety of biotechnologies, but cannabis has presented some challenges in uniformity and germination time, presumably due to the disinfection procedure. Disinfection and subsequent growth are influenced by many factors, such as media pH, temperature, as well as the types and levels of contaminants and disinfectants, which contribute independently and dynamically to system complexity and nonlinearity. Hence, artificial intelligence models are well suited to model and optimize this dynamic system. The current study was aimed to evaluate the effect of different types and concentrations of disinfectants (sodium hypochlorite, hydrogen peroxide) and immersion times on contamination frequency using the generalized regression neural network (GRNN), a powerful artificial neural network (ANN). The GRNN model had high prediction performance (R^2^ > 0.91) in both training and testing. Moreover, a genetic algorithm (GA) was subjected to the GRNN to find the optimal type and level of disinfectants and immersion time to determine the best methods for contamination reduction. According to the optimization process, 4.6% sodium hypochlorite along with 0.008% hydrogen peroxide for 16.81 min would result in the best outcomes. The results of a validation experiment demonstrated that this protocol resulted in 0% contamination as predicted, but germination rates were low and sporadic. However, using this sterilization protocol in combination with the scarification of in vitro cannabis seed (seed tip removal) resulted in 0% contamination and 100% seed germination within one week.

## 1. Introduction

For centuries, *Cannabis sativa* L. has been widely used around the world for various applications (e.g., textiles, food, cosmetics) [1]. These days, interest has been focused on medicinal and recreational facets, furthering commercial expansion. With Canada recently adopting the more globally appreciated view of cannabis, there exists an ever evolving, multi-billion-dollar industry focused on vegetative propagation [2]. Despite the reliance on clonal propagation, there is a continual need to germinate seeds to select new elite genotypes, perform pheno-hunting, as well as supporting breeding programs. To select new elite genotypes, plants are started from seed (technically achenes [3], but will be referred to as seed henceforth). During the vegetative phase of growth, a cutting is taken and maintained as a vegetative plant while the seedling is grown to maturity. Once the elite genotypes are selected, the cutting is then used as a source to propagate the clonal line. Maintaining the large population of cuttings during the phenotyping exercise represents a significant cost to producers and leaves the cutting derived mother plants exposed to insects and diseases. 

To address the issues of insect, disease, and viral infections in mother plants, many producers use plant tissue culture to ensure that they are starting with clean material. For this process, nodal segments are disinfected and established in culture, a time consuming and relatively expensive endeavor. A potential alternative to the traditional approach is to first establish the seed in tissue culture. Once the seedlings are established and multiplied, micropropagated clones can be transferred into the growth facility and cultivated to maturity to identify elite genotypes. After selecting the elite genotypes, the in vitro parent material would be available for clonal propagation. This approach would greatly reduce the amount of space required for selecting new cultivars and provide a ready source of clean planting material once elite genotypes are identified. However, this approach requires an effective in vitro seed germination protocol with high germination speed and frequency. An efficient in vitro seed germination system would also support downstream biotechnologies (regeneration, transformation, etc.) in which seedling-derived tissues are preferred [4,5,6]. 

We previously reported the effect of different types and strengths of media in addition to carbohydrate types and levels as primarily important factors contributing to in vitro cannabis seed germination indices and morphological seedling traits [5]. Our results demonstrated that maximum germination percentage (82.67 ± 3.837%) was achieved with 0.43 strength mMS medium and 2.3% sucrose [5]. While the germination rate was over 80%, this was after 40 days of culture. Typically, the cannabis seed germinates within several days in the greenhouse/growth room, suggesting that something during the disinfection process was interfering with subsequent germination. To improve our previous protocol, we hypothesize that optimizing the disinfection protocol and seed scarification would increase the speed and frequency of seed germination. 

**Figure 1:**
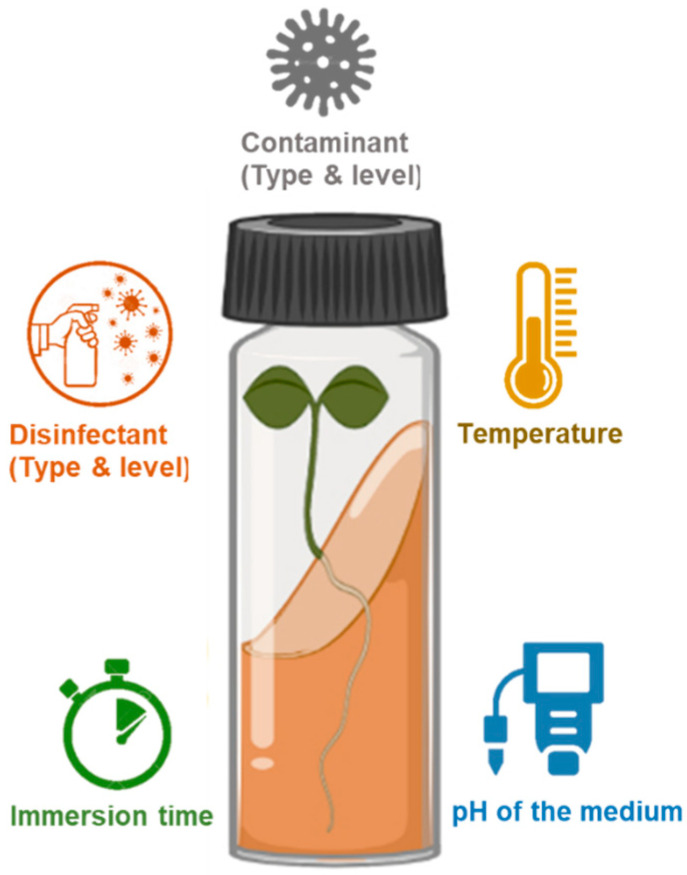
A schematic view of factors affecting the disinfection process.

As with most aspects of a tissue culture system, in vitro disinfection is a complex and non-linear process that is affected by numerous factors such as disinfectant and contaminant types and levels, media pH, immersion time, temperature, and their interactions (Figure 1) [7]. 

In the disinfection process, the concentration of disinfectants plays a conflicting dual role relating to contamination frequency and seed viability [8]. Higher disinfectant concentrations generally lead to a greater control over contaminants; however, lower seedling viability is often the trade-off [9,10]. Therefore, it is necessary to optimize the disinfection process. 

The disinfection process cannot be represented by a simple stepwise algorithm, especially when the datasets are highly imbalanced and noisy [10]. Therefore, artificial intelligence (AI) models combined with optimization algorithms (OAs) such as a genetic algorithm (GA) can be employed as an efficient and reliable computational method to interpret, forecast, and optimize this complex system [10,11,12,13,14]. This strategy (AI-OA) has been successfully used for modeling and optimizing different tissue culture systems, including in vitro decontamination, shoot proliferation, androgenesis, somatic embryogenesis, secondary metabolite production, and rhizogenesis [7,15,16]. Ivashchuk et al. [17] employed multilayer perceptron (MLP) and radial basis function (RBF) as two well-known artificial neural networks (ANNs) for modeling and predicting the effect of different disinfectants and immersion times for *Bellevalia sarmatica*, *Echinacea purpurea*, and *Nigella damascene* explant decontamination. They reported that both algorithms were able to accurately model and predict the disinfection process [17]. In another study, Hesami et al. [10] applied a hybrid MLP and non-dominated sorting genetic algorithm-II (NSGA-II) for the modeling and optimization of disinfectants and immersion times for chrysanthemum leaf segment decontamination. It was reported that MLP-NSGA-II had a high performance to predict and optimize the system [13]. A generalized regression neural network (GRNN) is another type of ANNs that has successfully been used for modeling and predicting different tissue culture processes [5,18,19,20]. Although there exist no reports using GRNN for the modeling and optimization of disinfection process, we previously showed that GRNN has a higher predictive performance than RBF, MLP, and the adaptive neuro-fuzzy inference system (ANFIS) for cannabis micropropagation [5,18]. Therefore, in the current study, we used GRNN-GA to model and optimize cannabis seed disinfection.

Mature seed germination can sometimes be more difficult than immature seed germination due to the increase in the seed coat’s impermeability and the accumulation of inhibitors during seed maturation [21]. Hence, dormancy breaking plays a critical role relating to the speed and frequency of seed germination due to morpho-physiological dormancy [22,23]. Although there are no reports on the effects of scarification on cannabis seed germination, the positive impact of dormancy breaking by scarification has previously been suggested in several plants, such as *Limodorum* [24], *Salvia stenophylla* [25], and legumes [22]. Based on this evidence, studying the effect of scarification on cannabis seed germination can pave the way for devising an in vitro seed germination protocol with high speed and germination frequency. The current study uses GRNN-GA to model and optimize cannabis seed disinfection, and investigates the effect of scarification on seed germination. By combining these procedures, a superior in vitro cannabis seed germination protocol that limits contamination while allowing high germination rates in a short timeframe was established.

## 2. Results

### 2.1. Effect of Different Disinfectants at Various Immersion Times on Contamination

Based on our results (Table 1), different contamination rates were observed in various disinfection treatments.

As shown in Table 1, sodium hypochlorite was more successful than hydrogen peroxide in controlling contamination. Also, different levels of sodium hypochlorite at 15 min of immersion resulted in no contamination (Table 1).

### 2.2. Data Modeling by Using GRNN 

According to our results, GRNN displayed an excellent performance for modeling and predicting contamination rates during in vitro sterilization (Table 2). Performance indices (RMSE and MBE) of the developed GRNN demonstrated that the obtained result was highly precise, and correlated in both training and testing sets (Table 2).

**Figure 2:**
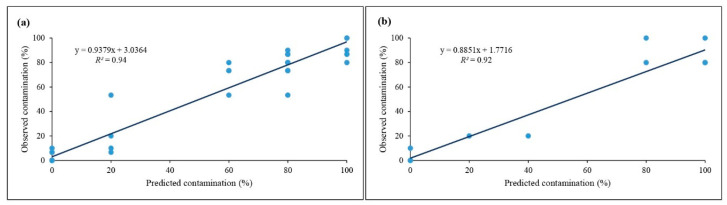
Scatter plot of observed data vs. predicted data of contamination percentage in (**a**) training and (**b**) testing sets.

Additionally, R^2^ was within the acceptable range for both training and testing sets, showing great prediction performance of the developed GRNN (Figure 2).

### 2.3. Optimization via GA and Validation Experiment

According to the optimization process (Table 3), 4.6% sodium hypochlorite along with 0.008% hydrogen peroxide for 16.81 min would result in no contamination. The results of the validation experiment verified that there was no contamination using this combination (Table 3).

### 2.4. Effect of Scarification on In Vitro Seed Germination 

**Figure 3:**
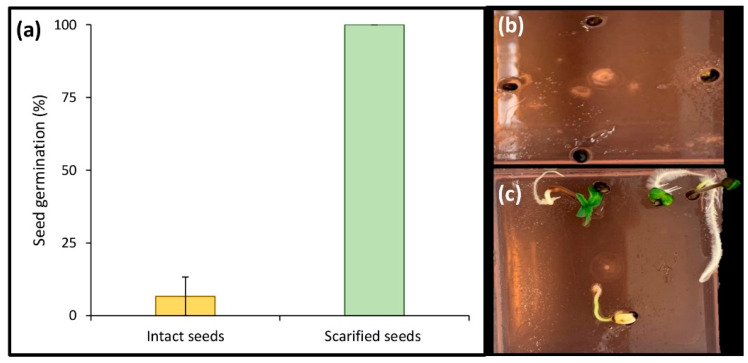
Effect of scarification on in vitro seed germination of cannabis after one week; (**a**) intact seeds vs. scarified seeds, (**b**) intact seeds, and (**c**) scarified seeds.

The seed scarification experiment (Figure 3) resulted in enhanced speed and frequency of in vitro germination of Finola seeds such that 100% germination was achieved within one week, while only 82.7 + 0.67% germination was observed in intact seeds (un- scarified seeds) after 40 days in our previous study. Moreover, we assessed the efficiency of the developed protocol on 10 drug-type cannabis genotypes (i.e., Bubba Island Kush, Glueberry OG, Critical Orange Punch, Frisian Dew, Banana Blaze, Blueberry, Durban Poison, Skunk #1, Passion #1, and Strawberry Cough; Dutch Passion, NL). The results showed more than 90% germination in these genotypes within one week when scarified.

## 3. Discussion

In vitro seed germination of cannabis has great potential to improve the efficiency of elite cultivar selection, pheno-hunting, phenotyping, and to support various in vitro culture methods as initial explant materials [5]. In orthodox seeds, germination typically initiates with the passive uptake of water by the dry mature seed, and terminates with radicle protrusion through the seed envelope [23]. Different abiotic factors (e.g., temperature, light, medium composition, sterilization procedures, and scarification) affect seed germination, mainly through regulating the signaling and metabolism pathways of abscisic acid (ABA) and gibberellic acid (GA) [21]. Although cannabis seeds easily germinate within several days under greenhouse or field conditions, in vitro cannabis seed germination tends to be more difficult, with lower germination rates spread over a longer period of time. The cause of this difference is unknown, but is likely related to the disinfection protocol that may stress the developing embryo or potentially eliminate microbes that play a role in the germination process. As such, optimizing sterilization and scarification protocols can be considered the two most important procedures for successful in vitro seed germination [25].

The surface sterilization of initial source material, including seeds, is a prerequisite for the success of the culture [10]. Therefore, it is vital to optimize the sterilization protocol while allowing it to remain simple, cheap, environmentally friendly, and efficient [8]. Although various disinfectants and immersion times can be employed to sterilize the explants, each species and even explant type necessitates a particular sterilization protocol [10]. The hybrid of machine learning—optimization algorithm procedures offer promising computational methodology that is well suited to model and optimize in vitro culture systems such as sterilization [10]. 

Based on our results, GRNN-GA accurately predicted and optimized the in vitro surface sterilization of cannabis seed. According to the optimization process through GRNN-GA, 4.6% sodium hypochlorite along with 0.008% hydrogen peroxide for 16.81 min would result in no contamination. Similar to our results, previous studies showed that sodium hypochlorite was more successful than hydrogen peroxide in controlling contamination [10,25]. Additionally, the results of the validation experiment confirmed no differences between the optimized predicted and observed results, showing the robustness of GRNN-GA. In line with our results, previous studies showed that GRNN-GA can be considered a reliable computational method with high prediction performance for the modeling and optimizing of in vitro culture systems [5,18,19,20]. 

**Figure 4:**
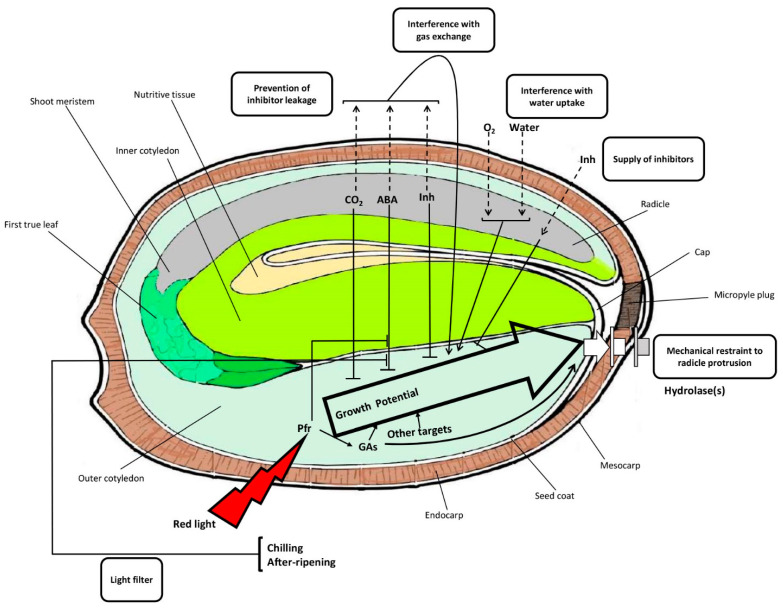
A schematic representation of potential interactions between the embryo and envelopes regulating cannabis seed germination and dormancy. GA: gibberellic acid; Pfr: far-red light photoreceptor phytochrome; ABA: abscisic acid; Inh: inhibitor.

While the optimized seed disinfection protocol resulted in 0% contamination, germination was still slow and sporadic. The second experiment was performed to evaluate the effect of scarification on the speed and frequency of in vitro seed germination. The speed at which in vitro cannabis seeds germinate is remarkably slow in comparison to field germination. One possible explanation for this difference is the presence of different microbes (e.g., bacteria) that aid in the digestion of the seed coat or micropyle plug, thereby facilitating higher rates of imbibition, and thus, higher/quicker field germination rates. Since micropropagation is performed in sterile conditions, it seems that an additional step (i.e., seed scarification) should be considered to achieve a high germination rate [25]. When a viable seed is not able to germinate under appropriate conditions, the seed is considered to be dormant [26]. While cannabis seed is generally not thought to exhibit dormancy, it would appear that under these unusual circumstances, it demonstrates some physical dormancy. Following water uptake by the quiescent, dry, mature seed, germination occurs once the embryo can prevail over the constraints imposed by the testa and associated tissues [27]. As shown in Figure 4, the main constraints exerted by the covering seed structures include (i) the mechanical prevention of radicle protrusion, (ii) water uptake interference, (iii) interference with gas exchange, especially carbon dioxide and oxygen (iv) light filtration, and (v) inhibitor leakage restraint from the embryo [26,27,28,29,30]. The removal of the seed coat had an important role in breaking seed dormancy, which ultimately resulted in higher germination speed and frequency. Our results showed that seed scarification by removing the seed tips significantly increased the speed and frequency of seed germination. In line with our results, Pérez-Jiménez et al. [31] reported that seed scarification (i.e., removal of the seed coat) significantly increased the in vitro seed germination of *Prunus persica* L. Batsch. For the first time, we have demonstrated this method with respect to the micropropagation of cannabis. 

## 4. Materials and Methods

### 4.1. Sterilization Procedure

Industrial hemp seed (*Cannabis sativa* cv. “Finola”; CSGA No.1 Certified seed, Lot #: 1908-18637-17-KKF-01) was employed in the first phase of this study. Different sterilants (sodium hypochlorite and hydrogen peroxide) at various immersion times (Table 1) were used for controlling contamination. The disinfection experiment was performed based on a completely randomized design with a factorial arrangement with 3 replications, each replication containing 5 seeds. For in vitro seed germination, the treated seeds were cultured in a previously optimized medium [5]. All media had 0.6% agar (Thermo-Fisher Scientific, Waltham, MA, USA) and the pH of the media was adjusted to 5.8 before autoclaving for 20 min at 120 °C. Thirty mL of media were poured into a Magenta GA7 box (Fisher Scientific, Hampton, NJ, USA). All culture boxes were placed in the growth chamber at 25  ±  2 °C under 16-h Photoperiod with 40  ±  5 μmol m^−2^ s^−1^ light intensity. Light was provided by multi-spectrum LEDs emitting only photosynthetically active radiation (400–700 nm). The obtained data was then used for modeling and optimizing the sterilization process using machine learning methods. 

### 4.2. Modeling Procedure

**Figure 5:**
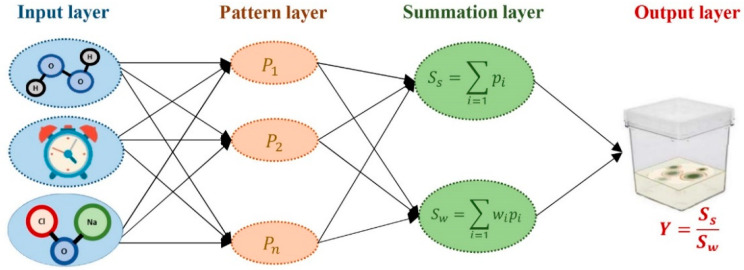
A schematic representation of generalized regression neural network (GRNN) used in this study.

In the current study, GRNN was used to develop a predictive model for contamination rate (Figure 5). To construct the model, sodium hypochlorite, hydrogen peroxide, and immersion time were considered as inputs, while the contamination rate was considered as the output (Figure 5).

Different performance criteria including Root mean square error (RMSE), mean bias error (MBE), and the coefficient of determination (R^2^) were used to assess the efficiency of the developed predictive model.

### 4.3. Optimization Procedure and Validation Experiment

**Figure 6:**
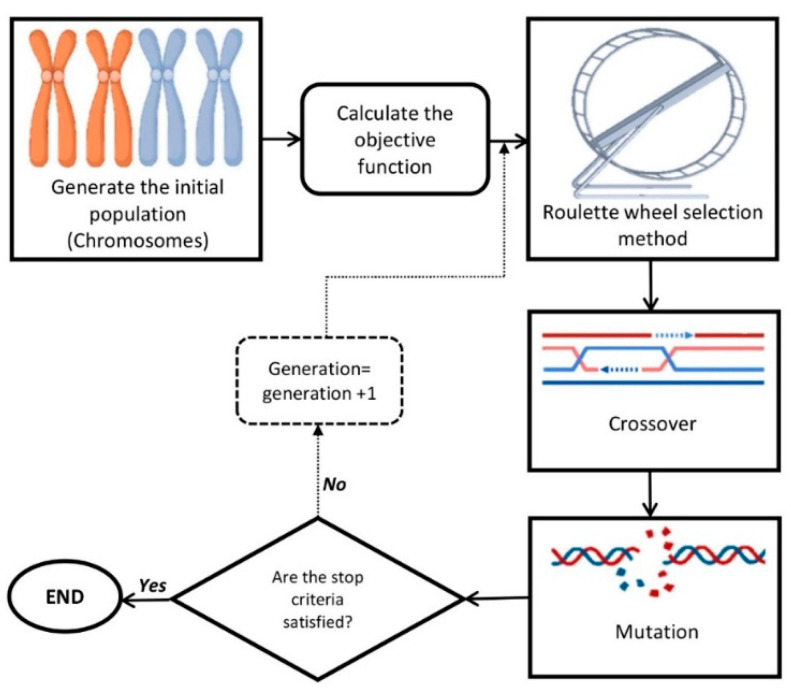
A schematic representation of genetic algorithm (GA).

After data modeling, the developed GRNN model was linked to a GA (Figure 6) to find the optimal level of sterilants and immersion time for minimizing the contamination rate. 

In this study, initial population, generation number, mutation rate, mutation function, selection function, cross-over fraction, and cross-over function were, respectively, considered as 200, 1000, 0.05, uniform, Roulette Wheel, 0.6, and Two-point crossover.

To assess the performance of the developed model, the predicted-optimized result of GRNN-GA was experimentally evaluated with 3 replications, each replication containing 4 seeds.

### 4.4. Scarification Procedure

**Figure 7:**
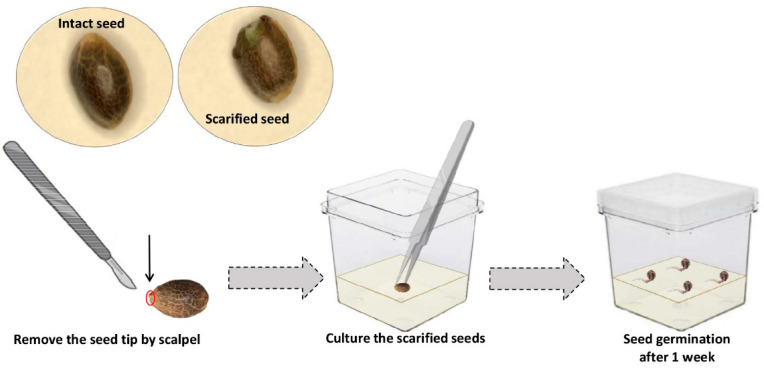
A schematic representation of the scarification methodology for in vitro cannabis seed germination.

To assess the effect of seed scarification, the micropyle and its surrounding tissue were removed by scalpel, without injury to the embryo (Figure 7). This experiment was performed based on a completely randomized design with two treatments (scarified seeds and intact seeds) with three replications. Each replication contained four seeds.

To assess the efficiency of the developed protocol, 10 drug-type genotypes of cannabis (i.e., Bubba Island Kush, Glueberry OG, Critical Orange Punch, Frisian Dew, Banana Blaze, Blueberry, Durban Poison, Skunk #1, Passion #1, and Strawberry Cough) were employed, and an in vitro seed germination rate on these genotypes was studied.

## 5. Conclusions

The present study was performed to establish an efficient in vitro seed germination protocol for *Cannabis sativa*. This was accomplished by optimizing an in vitro sterilization protocol for cannabis seeds using GRNN-GA, and to assess the effect of seed scarification on the speed and frequency of in vitro germination. The GRNN-GA model was able to precisely predict and optimize the disinfection process, but germination was still slow and sporadic. The result of the scarification experiment showed that seed scarification resulted in the reduction of in vitro germination time, while enhancing germination rate. Although we tested our protocol on different cannabis genotypes, future studies can evaluate the efficiency of the developed method on additional genotypes and further study the underlying mechanisms involved in seed dormancy and scarification in cannabis. Furthermore, we also suggest other methods, such as using sulfuric acid to evaluate scarification for in vitro cannabis seed germination; this development would improve the efficiency of the system. Due to the recalcitrant nature of *Cannabis* to in vitro culture, in addition to the much-needed refinement of in vitro seed disinfection techniques, the research presented offers opportunity to prepare micropropagated specimens with high efficiency. Our protocols can be implemented to reduce contamination and increase the germination rate of large-scale pheno-hunting and breeding programs for this economically important crop.

## Figures and Tables

**Table 1:** Effects of sodium hypochlorite and hydrogen peroxide (H_2_O_2_) concentrations and immersion times on contamination percentages of cannabis seeds.

Sodium Hypochlorite (%)	H_2_O_2_ (%)	Time (min)	Contamination (%) ± SE
0	0	0	100.0 ± 0.00
0	10	5	86.7 ± 6.67
0	10	10	86.7 ± 6.67
0	10	20	86.7 ± 6.67
0	20	5	86.7 ± 6.67
0	20	10	86.7 ± 6.67
0	20	20	73.3 ± 6.67
0	30	5	86.7 ± 6.67
0	30	10	80.0 ± 11.55
0	30	20	73.3 ± 6.67
5	0	5	53.3 ± 17.64
5	0	10	26.7 ± 6.67
5	0	15	0.0 ± 0.00
10	0	5	6.7 ± 6.67
10	0	10	0.0 ± 0.00
10	0	15	0.0 ± 0.00
15	0	5	6.7 ± 6.67
15	0	10	0.0 ± 0.00
15	0	15	0.0 ± 0.00

**Table 2:** Performance criteria of generalized regression neural network (GRNN) for contamination rate during cannabis seed disinfection.

Criteria	Training Set	Testing Set
Histogram	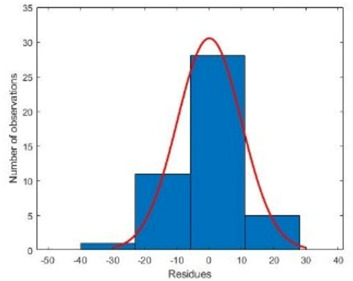	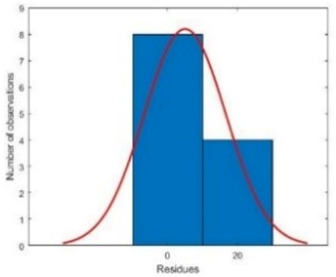
R^2^	0.938	0.918
RMSE	9.888	12.247
MBE	−2.250	−5.000

R^2^: Coefficient of determination; RMSE: Root mean square error; MBE: Mean bias error.

**Table 3:** The results of optimization process via genetic algorithm (GA) and validation experiment.

Optimal Level of Input Variables	Predicted Contamination (%)	Contamination (%) in Validation Experiment
4.6% sodium hypochlorite + 0.008% hydrogen peroxide for 16.81 min	0	0 ± 0.0

## Data Availability

All relevant data are within the paper.
